# Dog Demography, Animal Bite Management and Rabies Knowledge-Attitude and Practices in the Awash Basin, Eastern Ethiopia

**DOI:** 10.1371/journal.pntd.0004471

**Published:** 2016-02-22

**Authors:** Rea Tschopp, Shiferaw Bekele, Abraham Aseffa

**Affiliations:** 1 Armauer Hansen Research Institute, Addis Ababa, Ethiopia; 2 Swiss Tropical and Public Health Institute, Basel, Switzerland; Atlanta Health Associates, UNITED STATES

## Abstract

**Background:**

Rabies is a viral zoonosis that has been described in limited numbers of studies in Ethiopia at large and among pastoralists in particular. This study assessed dog demography, bite wound prevalence and management, potential risk factors of disease transmission and knowledge attitude practice towards rabies among urban dwellers, pastoralists and health workers in Awash, Eastern Ethiopia.

**Methodology:**

Information was collected by means of structured questionnaires and interviews and through medical and official records from the Agricultural and Health bureaus.

**Principal Findings:**

Respondents totaled 539 (471 urban, 49 pastoralists, 19 medical). Dog(s) were owned in 33% urban and 75.5% pastoralist households respectively. Mean dog number per dog owning household was 1.50 (95%CI: 1.40–1.60) in urban and 2.05 (95%CI: 1.51–2.60) in pastoralists sites. Human Dog Ratio in Metahara was 4.7:1. No bite wounds records were kept in medical facilities, where staff recalled around 100 bites per year, 2/3 being in adults. Over 90% of the respondents claimed knowing rabies but up to 79.2% pastoralist did not know how dogs acquire the disease; 37.3% urban and 23% pastoralist did not know the symptoms of rabies in dogs; 36% urban and 44% pastoralists did not know rabies symptoms in people. Eighty percent of pastoralists did not know that the disease was fatal in people if untreated. Over half (58.7%) of pastoralist respondents go to traditional healers if bitten, despite a health extension worker program in place in the study area. Knowledge gaps were also shown amidst medical staff.

**Conclusions:**

The study highlighted overall poor disease knowledge, severe under-reporting of human rabies cases, lack of record keeping and poor collaboration between the public and animal health sectors and communities in rabies control.

## Author Summary

Rabies is a fatal viral disease of animals and people. People usually get infected via bites from an infected animal (e.g. dog). Post exposure prophylaxis (PEP) has to be initiated immediately after bite wounds of suspected rabid animals in order to avoid fatalities. The situation of rabies is poorly known in Ethiopia, particularly in the pastoral context. We conducted questionnaire surveys in urban, pastoral and medical health worker communities around Awash National Park (Ethiopia) in order to capture information on dog demography, bite wound prevalence and management, potential risk factors of disease transmission and knowledge- attitude- practice (KAP) towards rabies among these communities. Disease knowledge was generally poor. Dog demography varied depending on the community which would affect control strategies. Health facilities did not keep bite records and there was poor recording and reporting of rabies cases. Delivery of PEP was inadequate. Communication and collaboration between the public and animal health sector was poor to inexistent regarding reporting and control of rabies cases.

## Introduction

Rabies is a viral zoonotic neglected disease caused by a negative stranded RNA virus from the Genus *Lyssavirus* [1].

Although a wide range of animals can become infected and transmit the disease, only mammals from the *Carnivora* and *Chiroptera* (bats) Order act as reservoir for the disease [2]. Domestic dogs are considered to be the main source (>90%) for human rabies in Africa [3, 4]. Once the symptoms have appeared, the disease ends almost always fatally. Transmission to people occurs predominantly via infected animal bite or scratch as well as via their saliva through mucosa and broken skin [3].

Therapy has to be initiated immediately and relies on Post-Exposure prophylaxis (PEP), which consists of rapid and thorough washing of the wound, completion of post-exposure vaccination schedules plus inoculation with rabies immunoglobulin (RIG) for severely exposed bite-victims.

The disease claims 24,000 human deaths annually in Africa alone [4, 5]. Rabies burden in Tanzania was 4.9 human death/100,000 based on active surveillance data on bite incidence and 0.62 human deaths/100,000 when based on national bite statistics [4]. Ethiopia is thought to be a high-burden country for rabies [6]. However, hard data on dog demography and ecology as well as true rabies incidence in dog and people are lacking. Information is based on estimations and extrapolations, small scale studies and limited record reviews [6–8]. The Ethiopian Public Health Institute (EPHI) is the only laboratory facility in the country to diagnose rabies and produce PEP. Ferni vaccines (adult sheep brain nervous tissue vaccines) were used at the time of this study, despite the WHO recommendation in 2006 to completely replace nerve tissue vaccine with cell-cultured based anti-rabies vaccines [9].A retrospective review from EPHI records between 2001 and 2009 showed that 1026–1580 patients per year in and around Addis Ababa were taking PEP and that the total fatality human cases was 35–58 per year [6]. A one year follow-up in Gondar based solely on clinical diagnosis revealed an incidence rate per year of 2.3/100,000 [8]. Rabies incidence however, is likely to be much higher considering the lack of accurate data and underreporting of cases [4, 5, 10]. Published data of rabies from rural areas of Ethiopia, including pastoralists are however entirely lacking.

The aim of this study was to try to gain a picture of the rabies epidemiology (prevalence, risk factors) at the animal-human interface in the Awash Basin. Dog demography, bite history and knowledge-attitude-practice (KAP) regarding the disease was assessed amongst pastoralists, urban dwellers and health workers by means of questionnaires and/or interviews. Constraints to rabies control and prevention in the study area are discussed.

## Methods

### Study Sites

**Fig 1:**
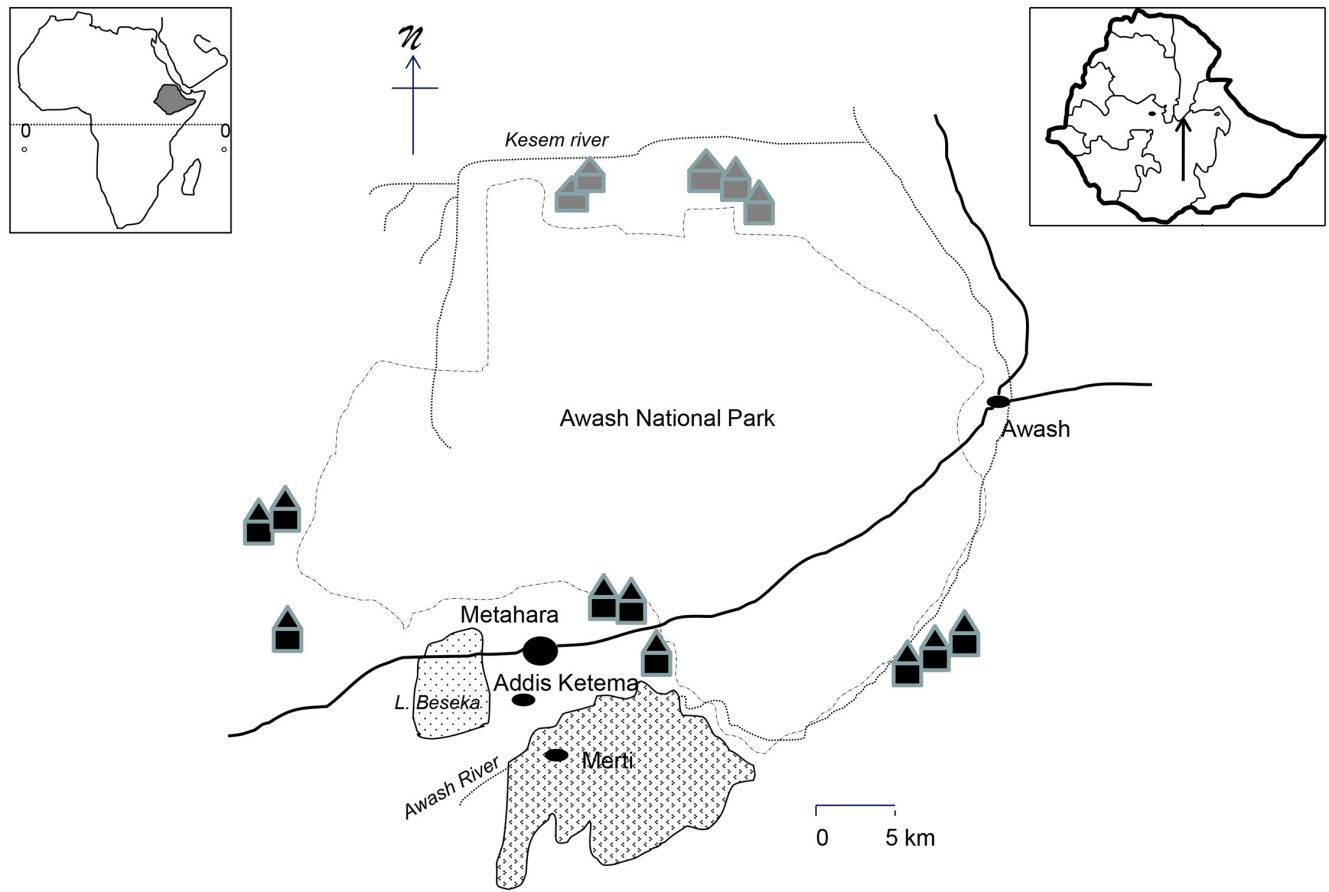
Sketch map of the study sites. Solid black circles representing towns, black solid huts representing Oromia pastoralist settlements and grey solid huts Afar pastoralist settlements (in total 7 clusters of pastoralist settlements). Inserts showing Ethiopia within Africa and the study sites within Ethiopia (tip of the arrow showing study site).

The study was carried out between February and June 2012 in Metahara, the administrative center of Fentale woreda (Oromia region) with a population of 25,670, its neighboring town Addis Ketema and Merti (Metahara Sugar Cane Plantation) as well as in the neighboring pastoral villages of the Oromia and Afar region (Fig 1). Urban dwellers were from various national ethnic backgrounds. Pastoralists were Ittu-Oromo, Kereyu-Oromo or Afar. Climate is semi-arid with bi-annual rainfalls. The area has an elevation ranging from 800 to 960 meter above sea level (National Meteorology Agency; Metahara Agricultural Bureau).

### Study Design

This cross-sectional study collected solely in depth interview/questionnaire data and no clinical samples. Questionnaires, with closed and open questions were translated into the local languages Amharic, Oromifa and Afarinia and back translated into English for consistency checks. The questionnaires were pre-tested in the study site. A trained interviewer administered all interviews in the local languages. Two sets of questionnaires were prepared, one for urban/ pastoralist dwellers and one for health workers. The first questionnaire attempted to capture information on dog population structure and husbandry as well as Knowledge Attitude Practice (KAP) of the interviewees regarding rabies. The question categories included: general information on the interviewee, questions related to dog husbandry and demography, contact of dogs with livestock and wildlife, questions related to bites (person bitten, bite location, bite wound treatment), questions related to disease awareness/attitude/practice (e.g. transmission to animal and people, symptoms and outcome in animals and people, source of disease knowledge, rabies therapy, bite wound therapy, rabies in livestock), and willingness to buy vaccine for dogs if available.

The second questionnaire aimed at assessing the knowledge of rabies amongst health workers. All types of medical facilities were included: health posts (N = 3), Health Centers (N = 1), private clinics (N = 3) and Merti hospital. Questions included: information on the interviewee and his work (age, sex, religion, work place, professional background, years spend in the profession), the treatment of bite wounds (kind of treatment, information on the bitten patient, bite location, number of bitten patient seen), whether or not he/she had rabies patients in the last 12 months and information on these patients, rabies knowledge (transmission and symptoms in animals and people), rabies therapy, and suggested intervention for rabies prevention in people.

The respondent was never offered answer options but had to talk freely on a question. The question was first asked in broader term to assess general knowledge and subsequently narrowed down to more detailed questions. This was to ensure no unwilling biased guiding from the interviewer.

In addition, verbal (oral evidence) and recorded data and general information on rabies were collected from both, the Health Bureau and the Agricultural Office in Metahara as well as from all medical facilities.

### Sample

We performed a non-probability sampling. A list of all pastoralist settlements was obtained from the Woreda Agricultural Bureaus of both Woredas; inclusion criteria were logistic feasibility (accessibility, security), proximity to the National Park, and willingness of pastoralists to participate in the interviews. All families of the chosen pastoral settlements and all medical staff present in the medical facility at the day of the interview were included in the study. For the urban study a house to house visit was made through the city and all families willing to answer the interview were included.

### Data Analysis

All data was entered into Microsoft Access tables and analyzed descriptively using Stata software (version 10.1, StataCorp, Texas, USA). A scoring between 1 and 3 was given to KAP parameters. For instance if a respondent could describe all main symptoms of rabies in dogs he would get a score of 1, if the respondent could not describe them but rather minor symptoms or remaining generally vague, he would get a score of 2. A score of 3 was attributed to respondents who claimed not knowing any symptoms. Pearson’s chi square statistics test was used to compare group differences for categorical variables in the KAP analysis as well as dog ownership by areas in Metahara. Associations and assessment of determinants for KAP were considered statistically significant if p<0.05.

### Ethical Consideration

The study received institutional ethical clearance from the AHRI/ALERT Ethical Review Committee (AAERC), number PO04/12. Heads of the Woreda Agricultural and Health Bureau in Metahara and Awash town were informed and permitted the project. All interviewees gave verbal informed consent.

## Results

Respondents totaled 539 (471 urban dwellers in Metahara, 49 pastoralists and 16 health workers, all medical facilities included). Amongst the health staff, all were nurses except for one health officer and 2 medical doctors. Nurses from health posts were also working as health extension workers.

### Dog Demography and Husbandry

Dogs totaled 236 in the 471 urban household and 76 among the 49 pastoralist households. Considering a population of 25,670 and 11,000 households (City Administration Metahara, annual census report 2011), the crude extrapolation for human dog ratio in Metahara was 4.7:1. Dogs were evenly represented throughout the city quarters (p: 0.138). Table 1 shows the demography and ownership of the dog population in Metahara and the pastoralist areas.

**Table 1:** Dog demography in urban (Metahara) and Oromia and Afar pastoralist study sites.

	Urban			Pastoralist
	Metahara	Oromia	Afar	Total
Number of HH* interviews	471	30	19	49
Number of HH with dog(s)	157 (33.3%)	24 (80%)	13 (68.4%)	37 (75.5%)
Total dog number (all HH)	236	35	41	76
Mean number dog/dog owning HH	1.5	1.25	3.15	2.05
95%CI for the mean	1.39–1.60	0.86–1.64	1.89–4.40	1.51–2.60
*Sex distribution (%)*				
Female	38.6	40	56	48.7
Male	61.4	60	43.9	51.3
Female: male ratio	1:1.6	1:1.5	1:0.8	1:1
*Age distribution (%)*				
Puppy (< 1year)	30.5	22.8	29.3	26.3
Adult (> 1 year)	69.5	77.2	70.7	73.7
Puppy: adult ratio	1:2.3	1:3.4	1:2.4	1:2.8

*HH: household

Dog ownership was not influenced by religious background (p: 0.12); 112/285 (39.3%) and 77/209 (36.8%) owned dog(s) among orthodox Christian and Muslim respondents respectively. Dog ownership differ statistically between urban and pastoral residents (p<0.001) (Table 1).

All dogs were kept as guard dogs and/or for livestock protection. None had received preventive rabies vaccination, was neutered/castrated, received veterinary care or had collars on. Puppies were never intentionally killed but kept or given away. They were all fed with left-overs from human consumption and/or left to roam free for food. The majority of the dogs were free-roaming during the day (stated by N = 114/157 (72.6%) urban and N = 33/37 (89.2%) pastoralist households) as well as during the night (N = 90 (57.3%) urban and N = 31/37 (83.8%) pastoralist households).

Dogs had regular contact with livestock in 94.3% and 94.6% of the urban and pastoralist households. Two urban and 14 (37.8%) pastoralist households had their dogs going regularly into the nearby National Park. Direct regular contact between dogs and wildlife was observed by N = 88/157 (56%) and N = 28/37 (75.7%) urban and pastoralist respondents respectively. Main wildlife species reported were hyenas, jackals and less often rabbits, monkeys and leopards.

### Bite Wounds and Treatment

None of the 471 urban and 9 (18.4%) pastoralist respondents experienced bite wounds in his/her household in the last 5 years. Among the pastoralists 1 was a child under 5 years, 5 were children between 5 and 15 years and 3 were adults. Bites were recalled to be located in the foot and leg (N = 7) and arm and hand (N = 2). Four out of the 9 bitten patients flushed the wound with water and soap while the others went to a medical facility in town. When bitten by an animal, 58.7% of the pastoralists, as opposed to 1% of the urban responded that they would go to traditional healers. The rest of the interviewed pastoralists either do wound cleaning themselves or go to medical facilities. Ninety-nine percent of the urban interviewees would go directly to a medical facility.

Bite wound information (e.g. patient age, sex, bite location, severity of bite) was not recorded in any of the medical facilities assessed. Overall, interviewed staff recalled 7 bites that they personally treated in the last 12 months, 2 from hyenas and 5 from dogs. The bite locations from dogs, as recalled, were most often in the legs followed by feet, arms and rarely the face. Hyenas on the other hand bit in the face. Total number of bite patients recalled from all health facilities was estimated to be around 100 per year, of which approximately 75% were adults. None of the health posts did treat patients with bite wounds and referred them directly to Health Centers or private clinics. Reasons given were the unavailability of water, soap and other disinfectant and medical supply. In the other visited facilities, all treating staff used disinfectants such as providone iodine to treat bite wounds as well as antibiotics. Initial flushing with water and soap/detergent has been done by 2 out of 16 respondents.

### Knowledge-Attitude-Practice

#### Urban and pastoralist respondents

The large majority of respondents claimed knowing rabies (98.5% urban; 91.8% pastoralists). Local names given were, in town, *Ebd wusha*, *Ebdat*, *Dhukuba saree maraate* and in the villages, *saree maratu*, *dukha saree*, *marat*. They all referred to “madness” in dogs. The majority of the respondents heard about the disease from the family unit (N = 429 (92.1%) in urban households; 86.7% in pastoralist households). The rest of the pastoralist respondents heard about it from the community at large or during their transhumance with livestock. Among urban respondents, N = 27/471 (5.8%) heard about it at school and N = 6/471 (1.8%) through the media (TV, radio).

Rabies transmission to dogs and to people as stated by urban and pastoralist respondents is shown in Table 2. Roughly a quarter of urban and 79.2% of pastoralists did not know how dogs acquire the disease.

**Table 2:** Transmission routes of rabies to dogs and to people as described by medical staff, urban and pastoralist respondents (total N = 539), shown as percentage.

	Medical staff	Urban			Pastoralist
		Metahara	Oromia	Afar	Total
Number of interviews	16	471	30	19	49
Number of respondents knowing rabies	16	464	28	17	45
***Rabies transmission to dog (%)***	ND				
Bites		0.2		3.6	2
Unspecific from other dogs		33.6		14.3	8.3
Specific from jackals		2.1			
Water shortage/long drought		39.6			
Food (rotten, contaminated)		0.4		10.7	6.2
Wind/air			5.5	7.1	6.2
Don't know		23.7	94.5	46.4	79.2
***Rabies transmission to people (%)***					
Animal bite	100	92	92.6	88.2	95.6
Saliva through broken skin	62.5	0.4			
Saliva contaminated objects	6.25	0.4			
Wind/air			3.6	5.9	2.2
Dog fur					2.2
Contact (unspecific) with dogs		5			
Don't know		2.4	3.6	11.8	6.6

Symptoms of rabies in dogs and in people as well as the outcome of a rabid patient as perceived by the respondents are listed in Tables 3 and 4 respectively.

**Table 3:** Rabies symptoms in dogs as perceived by the 539 respondents (shown as percentage).

	Medical staff	Urban			Pastoralist
		Metahara	Oromia	Afar	Total
Number of interviews	16	471	30	19	49
Number of respondents knowing rabies	16	464	28	17	45
Change of behavior	37.5		7.1		4.2
Restlessness/disorientation		11.6	42.8	22.2	33.3
Aggressivity, biting objects and people	75	10.8	28.6	5.6	18.8
Roaming, uncontrollable	25	11.6			
Salivation	75		14.3		8.3
Stop drinking	43.7				
Stop eating	62.5		7.1		4.2
Deep labored breathing	6.2				
Tail between the legs	50		7.1		4.2
Paralyzed tongue (hanging out of mouth)	18.7		3.6		2
Weight loss			7.1	61.1	27
Non-stop barking/crying	18.7	5.2	21.4	16.7	18.8
Fever	6.2				
Collapsed abdomen	6.2				
Normal eating and drinking	6.2				
Mental problems		10.3			
Unspecific sick (no appetite, fever)		25			
Coughing			3.6		2
Prostrated alone at home			7.1		4.2
Don't know the symptoms		37.3	21.4	27.8	23

**Table 4:** Rabies symptoms and disease outcome in people as perceived by the 539 respondents (shown as percentage).

	Medical staff	Urban			Pastoralist
		Metahara	Oromia	Afar	Total
Number of interviews	16	471	30	19	49
Number of respondents knowing rabies	16	464	28	17	45
***Rabies symptoms***					
Fever	37.5				
Hyperactive/excited	6.2				
Hydrophobia	37.5				
Muscle paralysis	6.2				
headache	12.5	1.5			
Confusion/agitation	6.2				
Abnormal behavior	37.5				
Paralysis	6.2				
Salivation	18.7				
Acting like a dog	25		53.6	22.2	37.5
Biting	37.5				
Barking/screaming	68.7	1.7			
Mad	6.2	38.1	10.7		6.2
Vomiting	6.2		3.6		2
Running around			10.7		4.2
Sweating		0.2			
Development of wounds		1			
Unspecific sick		21.1			
Not eating	31.2		10.7		4.2
Aggressive	31.2				
Fainting/unconsciousness	12.5				
Nausea	6.2				
Stiff neck	6.2				
Convulsion	6.2				
CNS symptoms	12.5				
Change body color (turn dark)			7.1		4.2
Salivation			10.7		4.2
Don't know the symptoms		36	25	77.8	43.8
***Rabies patient outcome***					
Death	100	52.4	32.1		20
Madness		45			
Don't know		5.6	75	100	80

The majority of respondents knew that livestock can acquire rabies (88.8% urban, 72.9% pastoralist respondents) and that livestock can transmit rabies to people (88.4% urban, 66.7% pastoralist).

Among the urban interviewees, 99% were willing to pay annually for a dog vaccine if it was available (mean price: 241.5 Ethiopian Birr (ETB); SD: 160.5 ETB). In the pastoralist villages, 73.5% were willing to do so (mean price: 6.4 ETB; SD: 28.8 ETB).

Owning dog(s) was a determinant for knowledge of rabies symptoms in people and dogs (p:0.001) and knowing that livestock can transmit rabies to people (p:0.004). Lifestyle (urban versus pastoral dweller) was a determinant for knowledge of rabies symptoms in people (p: 0.0001), how dogs acquire rabies (p:0.001) and that people can acquire rabies from livestock (p<0.0001). Other possible determinants such as educational background, socio-economic status and gender were not included in the survey.

#### Health workers

One medical doctor and 15 nurses were interviewed. The majority were Muslim (N = 12/16; 75%) with the rest being equally catholic or orthodox Christians. Seven (43.7%), 5 (31.3%) and 4 (25%) were aged between 20–29 years, 30–39 years and 40 years old and over respectively. The majority of respondents had been in the profession between 1 and 3 years (56.3%), whereas only 1 was freshly graduated, 4 (25%) had been working between 4 and 9 years and 2 (12.5%) had been employed over 10 years. Responses were not affected by staff age or years spent in the profession. All respondents claimed knowing rabies. Among them, N = 12/16 (75%) said it was caused by a virus whereas N = 4/16 (25%) thought it was a bacterial disease. Transmission routes of rabies to people as perceived are shown in Table 2. Symptoms of rabies in dogs and people as perceived by health workers are listed in Tables 3 and 4 respectively. Knowledge of rabies symptoms was poorer in private clinics than in government facilities. Also, two respondents from private clinics said they would generally treat patient with fever first for malaria, even if a bite wound was present, since malaria is highly prevalent in the area.

Incubation time was thought to be between 10 and 42 days and symptoms durations between 2 and 30 days depending on the respondents. One medical respondent did not know about incubation and symptoms duration. Cure for rabies was said to be good disinfection of the bite wound (N = 1), injection (N = 1; but did not know what exactly), and PEP (N = 14). Asked about the number of PEP injections needed by a patient, 2 medical staff did not know, 2 said 2 injections, 2 said 14 injections, 4 said 17 injections and 1 said 18 injections. A quarter (26.7%) of the medical staff did not know if PEP was available in Metahara or elsewhere. How and if rabies is diagnosed in dogs was unknown by respectively 68.7% and 25% of the health workers interviewed.

That livestock can acquire rabies was unknown by 25% of the respondents. Transmission route from livestock to people was thought to be through bites (43.8%; N = 7), saliva (25%; N = 4), milk and/or meat consumption (43.7%; N = 7) while 37.5% (N = 6) did not know the route. Possible rabies transmission though bats was known only by 2 respondents.

Preventive measures suggested by the medical staff respondents were, in order of importance: preventive dog vaccination (56.2%; N = 9), people awareness for the disease (50%; N = 8), early disease detection so that patient can start PEP as soon as possible (25%; N = 4), total elimination/killing of stray dogs (25%; N = 4) and improved dog husbandry (e.g. not letting dogs stray) (25%; N = 4).

### The 2012 Outbreak-Known Facts

This study was done during a rabies outbreak, which started in the rural areas South-West of the Awash National Park in May 2012. A strychnine poisoning campaign, in a bid to stop the disease spread in the urban areas, killed in a couple of days 275 dogs (personal communication, Merti hospital). The pastoralist communities stated that many people had been bitten and had died of rabies during the outbreak. Exact numbers, however, were unavailable. The Agricultural Bureau stated (recalls only as no written records) that the outbreak lasted 2 months and that 11 livestock at least had died of rabies and 4 people were on PEP therapy. The Health Bureau officially recorded 2 patients undergoing PEP treatment and stated the outbreak did last 1 week only. In May only, Merti hospital on the other hand, treated 32 patients for bite wounds and 15 patients with PEP.

Dogs that are biting people are not further diagnosed as whether they have rabies or not. The decision to start PEP treatment on a bitten patient relies solely on the decision of the health worker.

## Discussion

### Dog Population

Control of zoonosis and prevention of human cases is usually most cost-efficient by controlling the disease in the animal reservoir [11]. Knowledge of the dog population structure, dynamics and ecology is, however, an essential pre-requisite to achieve proper required preventive vaccination coverage in the dog population (critical vaccination coverage varies with animal density), not to waste scarce financial and logistic resources and avoid large-scale campaign failure [12, 13]. In Ethiopia, published data on dog population number, structure and dynamics is lacking. In our study, 33% of the urban (grossly 1 dog per 5 people) and 75.5% of the pastoralist households kept dogs, regardless of religious background. Despite it being a small geographical study area, it could be observed that dog ownership and demography differed between the urban and the rural households but also between the pastoralist groups (Afar versus Oromo). The observed sex-and age- based dog population imbalance is in line with previous findings [14,15]. Respondents in our study, with the exception of the Afar, generally preferred male dogs, a trend reflected in many developing countries [15]. Dogs were not intentionally killed, therefore a steady population increase would be assumed. However, in general, free roaming dogs have short life expectancy and 2/3 die in their first year [15]. In Kenya, a study showed that life expectancy for males was 3.5 years and for females 2.4 years [12]. In our study, veterinary care was inexistent and dog husbandry poor, factors likely to lead to high dog mortality.

A common perception of all respondents was the large number of stray dogs, hence un-owned dogs. This study did not investigate the number of stray dogs but we need to keep in mind they also contribute to human rabies. Pastoralists stated that they were coming from town. However, the majority of owned dogs were free-roaming (day and night time), and in constant quest for food since they receive only little left-overs at home. In addition, they were not wearing collars, thus easily mistaken for stray dogs. Studies have shown that in reality most dogs are owned [16,17]. This is an opportunity for health intervention campaigns since the fact that dogs have owners would facilitate regular vaccination campaigns in Metehara. To support this, the majority of the urban and pastoralist respondents stated that they would be willing to regularly pay for their dog’s vaccination if the vaccine was indeed available.

Large scale lethal poisoning with strychnine is a common preventive measure undertaken in dogs in Ethiopia. Four out of 16 health workers reported killing of stray dog as a means of eliminating dogs. Mass dog elimination, however, besides being unethical and hazardous to the environment, has been shown to be counter-productive as it will not affect the dog population size and will not stop the spread of rabies as dogs will engage in compensatory breeding and migrate into newly vacated territories, thus facilitating disease transmission [18,19]. Morters et al (2013) recently showed as well that rabies transmission is not density-dependent [20].

### Reporting and Record Keeping

Rabies is one of 20 reportable diseases in Ethiopia. Our study showed however, that rabies notification was poor compared to other diseases. The authors observed a severe discrepancy between the orally recalled rabies cases, the number of used PEP bottles logged into medical facility pharmacies, and the number of cases actually officially recorded and reported. Mainly adults were recalled to have been bitten. Generally, children are known to be more at risk for being bitten [9,21,22]. Our results raises serious concern as whether children were indeed less at risk or whether they were not brought to health facilities when bitten, thus showing that rabies in children is likely to be underreported and their treatments severely neglected in these communities.

Bite locations were most often in the legs followed by feet, arms and rarely the face. This picture differed from a study in Tanzania where dogs bit mostly hands and face [4]. The location of the bite and the wound severity (scratch versus deep skin penetration) is likely to affect the outcome into clinical rabies [4]. Our study unfortunately could not collect enough details on bite wounds. Bite records (location, severity, patient identification) were not kept in any of the assessed health facilities. All information on bite wounds was collected only through verbal recollection of health workers. The authors assume however that the wounds must have been severe for patients to come to health facilities considering the cost and time involved for patients. Reliable record keeping of bite wounds has been shown to be a useful epidemiological tool as a proxy for human exposure and rabies incidence in animals [2,10,23,24]. In our case, calculation of rabies cases—and exposure- incidences in people and animals was also impossible due to the lack of accurate record keeping, severe underreporting, lack of population census amongst pastoralists and the dog population in particular.

### Health Seeking Behavior

Pastoralists of the study area are likely not to go to a medical facility when bitten; reasons include distance to health facility/logistics, mistrust in the medical system, and poor knowledge of the disease fatal outcome. Pastoral respondent showed to be a determinant for poorer knowledge of rabies particularly for rabies in people. Despite the presence of an extension health worker system, over half (58.7%) of the respondents said they would go to traditional healers if bitten. It is estimated that the majority of human rabies deaths occurs in rural rather than in urban areas [10,25].

### Health Delivery and Disease Awareness among Health Workers

The main constraint to human rabies prevention in the study area was the quantitative lack of PEP, and its immediate inaccessibility (the vaccine is available in Adama, 134 km away from Metahara) thus delaying severely the start of the prophylaxis. From the moment a person was bitten to the start of PEP injections, delays of several days (up to 5 days) were not unusual, particularly if patients came from rural areas. Wound cleaning was rarely performed as first aid, neither by the patients themselves nor the medical staff, which is a behavior described in other developing countries in Africa and Asia [22, 26,27]. However, immediate flushing of a bite wound for 15 minutes with water and soap can be lifesaving, as the virus is mechanically removed from the site or is rendered unable to invade tissue [28]. Neglecting immediate wound flushing was shown to increase the risk of developing rabies by fivefold [21]. Health posts, that are often the first health facility patients, particularly pastoralists, would visit, were not offering this simple, cheap and important service. Hence, health posts and health extension workers visiting remote rural villages are in a unique position to initiate this procedure before transferring a patient to a larger facility that could start PEP, to educate people at large and pastoralists in particular about the importance of immediate wound cleaning, as well as the outcome of an untreated patient with rabies. However, this study also highlighted knowledge gaps about rabies among the health staff. Patients, particularly pastoralists who are not knowledgeable about the disease will rely on the health practitioner’s advice for adequate treatment, as whether PEP should be administered or not, as also seen in a study in Tanzania [22]. In our study, 25% of health staff did not know that livestock can transmit the disease to people. Hence, patients bitten by rabid livestock are likely not going to be treated for rabies. Also only 2 out of 16 health workers knew that bats can transmit rabies. The role of bats in carrying and transmitting rabies in Ethiopia is not known. However, its role should be mentioned in any future awareness programs. The fact that 25% of health workers thought that rabies is a bacterial disease raises the question as whether they thought PEP is an antibiotic and/or if they would treat the patient with antibiotics. Unfortunately we did not look further into this point. These results overall show an urgent need of improved training of health workers in rabies epidemiology and treatment.

On the other hand, the lack of diagnosis in dogs implies that likely a high number of patients are unnecessary treated, putting a strain on the already difficult logistics, economics and availability of PEP. Deressa *et al*. (2010) showed that only 10% of the dogs that had bitten people and were brought to EPHI for quarantine were actually rabid [6]. There are currently, new rapid and simple rabies diagnostic tests on the market that can be used directly under field condition, such as the Anigen rabies test, an immunochromatographic test (ICT) giving results within minutes, not requiring expertise nor special facilities/equipment and thus helping in the decision of PET use [29]. This would require, however, close collaboration and communication between the Agriculture Office, the Health Bureau, the veterinarians, the clinicians, and the communities. In our study, we could observe that collaboration and communication between the different stakeholders was poor to non-existent.

### Disease Awareness among Non-medical Respondents

This study highlighted overall poor disease awareness amongst non-medical respondents although over 90% stated knowing the disease. A high percentage of respondents, particularly pastoralists (79.2%) did not know how dogs acquire rabies. Water shortage, wind and consumption of rotten food were often given as reason. Over a third of respondents did not know the symptoms of rabies in dogs and in people. The fate of a rabid person was not known by the majority of the pastoralist respondents (up to 80%). This highlights that the seriousness of this fatal disease in people was poorly known.

In a country that lacks hard data on epidemiology, vaccines for dogs and PEP for humans, and that is tight by logistic and financial constraints to efficient medical rabies control, disease awareness takes an important place in disease prevention. Our study showed that family nucleus and the community particularly amongst pastoralists played a central role in passing down knowledge about the disease whereas school and media played a minor role. Urban respondents may have better access to media, but pastoralists gather a lot of information from the pastoral community during transhumance with their animals. The latter were also aware of the economic burden of the disease since livestock can be affected.

The use of media can be instrumental to increase rabies awareness in a population, similarly as was- and is being done with other diseases such as tuberculosis in Ethiopia; it targets large audience and can also help promoting responsible dog ownership.

The overall rabies knowledge amongst health practitioners was variable and showed some knowledge and diagnosis/intervention gaps, particularly among staff from private clinics. Rabies is still sometimes misdiagnosed for malaria.

This study was the first in its kind to look at a rabies situation in a defined study site from a holistic approach including as well the animal as human side, urban and pastoralist respondents, medical and non- medical people. The in-depth interviews as well as the analysis of official records gave a valuable insight in the many existing gaps on rabies knowledge, prevention and health delivery. These information are valuable before embarking in a control and or awareness program in the study area. The limitations of the study lay in the non-probability sampling method used and the small sample size as well as the limited information for KAP determinant analysis. Also all data on bites and rabies patients (animal and human) were solely based on recalls and not on official records calling for inevitable biases.

### Conclusions

Rabies is a 100% preventable disease but numerous challenges and constraints in Africa render its control and elimination difficult, hence relegating it to the neglected diseases [2, 30, 31]. In this study, it was observed that preventive dog vaccination was non-existent due to lack of vaccine availability at the time. In such a situation, disease awareness takes even more so, an important place in disease prevention as well in urban as in pastoral communities. However, this study highlighted overall poor disease knowledge and gaps in recognizing and treating rabies in people, the likelihood of severe under-reporting of cases, poor medical facility registries regarding bite wounds and rabies and a lack of collaboration between the animal and public health sectors. These factors are likely to hamper any future efficient rabies control campaign in the study area.
